# Supplementary material for: Iterative improvement in the automatic modular design of robot swarms
Source: PeerJ Comput Sci. 2020 Dec 7;6:e322. doi: 10.7717/peerj-cs.322 (PMC7924708; doi:10.7717/peerj-cs.322)
Supplement: Supplemental Information 3 [file peerj-cs-06-322-s003.zip › argos3/doc/api/standalone/a00569_source.html]

ARGoS: plugins/simulator/entities/gripper\_equipped\_entity.h Source File


- Main Page
- Related Pages
- Namespaces
- Classes
- Files

- File List
- File Members

# plugins/simulator/entities/gripper\_equipped\_entity.h

Go to the documentation of this file.

```
00001 
00007 #ifndef GRIPPER_EQUIPPED_ENTITY_H
00008 #define GRIPPER_EQUIPPED_ENTITY_H
00009 
00010 namespace argos {
00011    class CGripperEquippedEntity;
00012    class CEmbodiedEntity;
00013 }
00014 
00015 #include <argos3/core/simulator/entity/entity.h>
00016 #include <argos3/core/utility/math/vector3.h>
00017 #include <argos3/core/utility/math/quaternion.h>
00018 
00019 namespace argos {
00020 
00037    class CGripperEquippedEntity : public CEntity {
00038 
00039    public:
00040 
00041       ENABLE_VTABLE();
00042 
00043    public:
00044 
00050       CGripperEquippedEntity(CComposableEntity* pc_parent);
00051 
00063       CGripperEquippedEntity(CComposableEntity* pc_parent,
00064                              const std::string& str_id,
00065                              const CVector3& c_offset,
00066                              const CVector3& c_direction,
00067                              Real f_lock_threshold = 0.5f);
00068 
00073       virtual void Init(TConfigurationNode& t_tree);
00074 
00078       virtual void Reset();
00079 
00084       inline const CVector3& GetOffset() const {
00085          return m_cOffset;
00086       }
00087 
00093       inline const CVector3& GetInitOffset() const {
00094          return m_cInitOffset;
00095       }
00096 
00101       inline void SetOffset(const CVector3& c_offset) {
00102          m_cOffset = c_offset;
00103       }
00104 
00110       inline void SetInitOffset(const CVector3& c_offset) {
00111          m_cInitOffset = c_offset;
00112       }
00113 
00118       inline const CVector3& GetDirection() const {
00119          return m_cDirection;
00120       }
00121 
00127       inline const CVector3& GetInitDirection() const {
00128          return m_cInitDirection;
00129       }
00130 
00135       inline void SetDirection(const CVector3& c_direction) {
00136          m_cDirection = c_direction;
00137       }
00138 
00144       inline void SetInitDirection(const CVector3& c_direction) {
00145          m_cInitDirection = c_direction;
00146       }
00147 
00153       inline Real GetLockState() const {
00154          return m_fLockState;
00155       }
00156 
00162       void SetLockState(Real f_lock_state);
00163 
00169       inline Real GetLockThreshold() const {
00170          return m_fLockThreshold;
00171       }
00172 
00178       void SetLockThreshold(Real f_lock_threshold);
00179 
00187       inline bool IsLocked() const {
00188          return m_fLockState > m_fLockThreshold;
00189       }
00190 
00195       inline bool IsGripping() const {
00196          return (m_pcGrippedEntity != NULL);
00197       }
00198 
00204       CEmbodiedEntity& GetGrippedEntity();
00205 
00210       inline void SetGrippedEntity(CEmbodiedEntity& c_entity) {
00211          m_pcGrippedEntity = &c_entity;
00212       }
00213 
00217       inline void ClearGrippedEntity() {
00218          m_pcGrippedEntity = NULL;
00219       }
00220 
00221       virtual std::string GetTypeDescription() const {
00222          return "gripper";
00223       }
00224 
00225    private:
00226 
00228       CVector3 m_cOffset;
00229 
00231       CVector3 m_cInitOffset;
00232 
00234       CVector3 m_cDirection;
00235 
00237       CVector3 m_cInitDirection;
00238 
00240       Real m_fLockState;
00241 
00243       Real m_fLockThreshold;
00244 
00246       CEmbodiedEntity* m_pcGrippedEntity;
00247       
00248    };
00249 }
00250 
00251 #endif
```

---

Generated on 10 Jul 2018 for ARGoS by 
 1.6.1
